# Applicability of the Geographic Tongue Area and Severity Index among Healthcare Professionals: A Cross-Sectional Clinical Validation of a Newly Developed Geographic Tongue Scoring System

**DOI:** 10.3390/jcm10235493

**Published:** 2021-11-24

**Authors:** Bruna Lavinas Sayed Picciani, Lílian Rocha Santos, Thaylla Núñez Amin, Jonatas Daniel Rocha Santos, Sueli Carneiro, Jane Marcy Neffa Pinto, Joao Carlos Regazzi Avelleira, David Rubem Azulay, Heron Fernando de Sousa Gonzaga, Ronir Raggio Luiz, Arkadiusz Dziedzic

**Affiliations:** 1 School of Medicine, Fluminense Federal University, Niteroi 28625-650, RJ, Brazil; lilian_ei@hotmail.com (L.R.S.); thayllanunez@gmail.com (T.N.A.); 2 Federal Center for Technological Education Celso Suckow da Fonseca, Rio de Janeiro 20271-204, RJ, Brazil; jonatasdrs@yahoo.com.br; 3 Sector of Dermatology, Department of Medical Clinic, Rio de Janeiro Federal University, Rio de Janeiro 21949-590, RJ, Brazil; sueli@hucff.ufrj.br; 4 Department of Dermatology, Fluminense Federal University, Niteroi 24220-900, RJ, Brazil; janeneffa@gmail.com; 5 Institute of Dermatology, Santa Casa da Misericórdia, Rio de Janeiro 20020-022, RJ, Brazil; avelleira@uol.com.br (J.C.R.A.); drubazulay@gmail.com (D.R.A.); 6 Department of Dermatology, Medical School, UNIMAR, Marília 17519-030, SP, Brazil; herongonzaga@yahoo.com.br; 7 Institute of Public Health Studies, Rio de Janeiro Federal University, Rio de Janeiro 21941-598, RJ, Brazil; ronir@iesc.ufrj.br; 8 Department of Conservative Dentistry with Endodontics, Medical University of Silesia, 40-055 Katowice, Poland

**Keywords:** geographic tongue, psoriasis, geographic tongue area severity index, evaluation, validation

## Abstract

Geographic tongue (GT) is a chronic condition of unknown aetiology, with no defined parameters to establish the protocol for evidence-based management. Validation of a newly developed and proposed clinical index to assess the severity of GT could assist in its diagnosis, especially in cases associated with systemic dermatological diseases in the form of psoriasis. Objective: To verify the applicability of the geographic tongue area and severity index (GTASI). This involved healthcare professionals from different specialties to evaluate the usefulness of the GTASI in supporting GT classification, as well as the follow-up process. Methods: One hundred cases of previously diagnosed GT were initially evaluated by three independent, experienced researchers/clinicians to obtain a standardised classification baseline. Subsequently, nine cases of GT were selected, three cases for each category—mild, moderate and severe. These stages were professionally evaluated by 51 healthcare professionals from three groups: 17 dentists (33%), 22 oral medicine specialists (43%) and 12 specialist dermatologists (24%) during a cross-sectional survey. Results: The quantitative and qualitative assessment based on experts’ opinions in the cross-sectional survey demonstrated an acceptable, similar level of GT clinical diagnosis (*p* > 0.05), with coherence between the various groups of professionals critically appraising the GTASI. An apparent divergence was observed for the moderate GT category, as well as in the group of less experienced evaluators. Conclusion: Whilst the validation of GTASI applicability was successfully executed, the general dental practitioners, specialists in oral medicine and dermatologists were equally capable of correct GT diagnosis and appropriately rating its severity. These coherent results were especially replicated among the experienced clinicians. The validation of the newly proposed index confirmed its reliability as a feasible instrument in oral medicine, with the prospect of its wider implementation in clinical practice.

## 1. Introduction

Geographic tongue (GT) is classified as a chronic and immunologically mediated condition characterised by a lymphocytic response that generates epithelial atrophy, particularly of the filiform papillae. Although it has been described for decades, its etiopathogenesis remains unknown [1,2]. This oral lesion has been associated with systemic diseases, primarily psoriasis, and is considered an oral manifestation and soft tissue “marker” of the severity of some dermatoses [3,4,5].

Clinically, erythematous atrophic areas, often circumscribed by a slightly elevated white halo, are usually observed, with episodes of spontaneous remission, exacerbation and migration of the lesions [6,7,8]. Tentative diagnosis is driven mainly by clinical assessment. Tongue lesions are usually asymptomatic; however, in some cases, patients may report non-specific symptoms in the form of burning, pain or tingling sensations of the lesions [5,9]. Whilst there is no standardised protocol for clinical assessment and treatment of GT, it is essential to validate clinical parameters to precisely diagnose and adopt appropriate therapy, if required, depending upon the severity grading.

While recent studies have suggested that GT may manifest as an early oral marker of psoriasis onset and severity [4,5,8], it should be included in the criteria for the disease’s diagnostic, as well as its therapeutic definition, highlighting the importance of careful evaluation of GT by different healthcare professionals [10,11,12]. Because these reports indicate that GT is a potential oral manifestation of psoriasis (Table 1), the geographic tongue area and severity index (GTASI) was developed and proposed, based on cumulative clinical data and experts’ recommendations. It adapts an existing and already consolidated clinical index for psoriasis, i.e., the Psoriasis Area and Severity Index (PASI), based on the clinical aspects of this lesion [13]. It was recently suggested that the PASI should be implemented by experienced professionals and scholars as an assessment tool for GT and psoriasis diagnostics to verify its validity among different categories of health professionals.

The validation of clinical indices used for diagnostic purposes requires a complex, multi-level process to confirm the reliability of newly proposed classifications. This crucial stage requires various experts in specific fields and professions to test the applicability of novel clinical indices in various healthcare settings. While GT appears to be an oral manifestation of underlying systemic conditions, GT severity classification seems well justified to support early diagnosis protocol and clinical management. GTASI provides a standardized protocol for GT assessment and for the uniform interpretation of clinical characteristics.

Taking into consideration the lack of uniform classification of GT, including its severity grades, this aim of this study was to validate the reliability of the newly developed clinical index (GTASI) in assessing and classifying GT lesions based on the opinions of experts from three different specialties. In addition, the cross-comparative study was designed to identify variations/discrepancies in recognizing tongue lesions and validating the GTASI.

## 2. Materials and Methods

### 2.1. Geographical Tongue Area and Severity Index Scoring and Calculation

**Figure 1:**
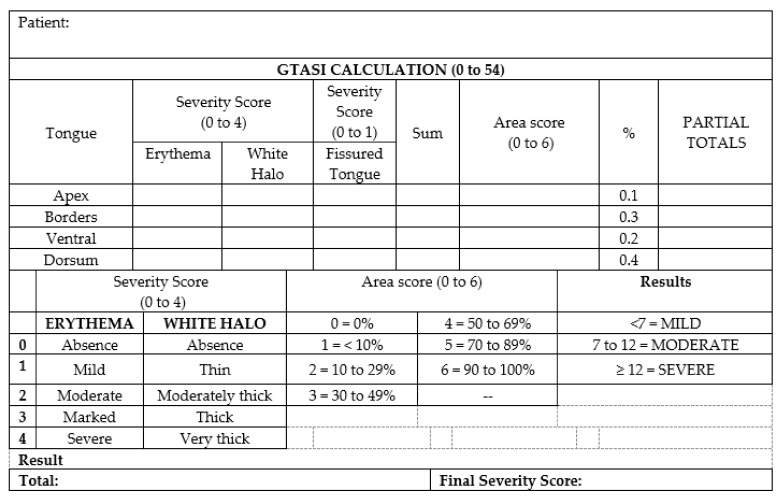
Geographic Tongue Area Severity Index calculation.

The prospective cross-sectional study was designed to validate the clinical application of the GTASI among health professionals who treat patients with GT most often, by utilising the GTASI [13]. In a previous study, Picciani et al. (2020) developed the GTASI index (Figure 1), which was applied in one hundred cases of GT by three researchers and scholars experienced in recognising oral lesions (oral medicine experts). The lesions were categorised into three groups based on their final score: 1–6 points were classified as mild, 7–12 points as moderate and above 12 points as severe [13]. 

**Figure 2:**
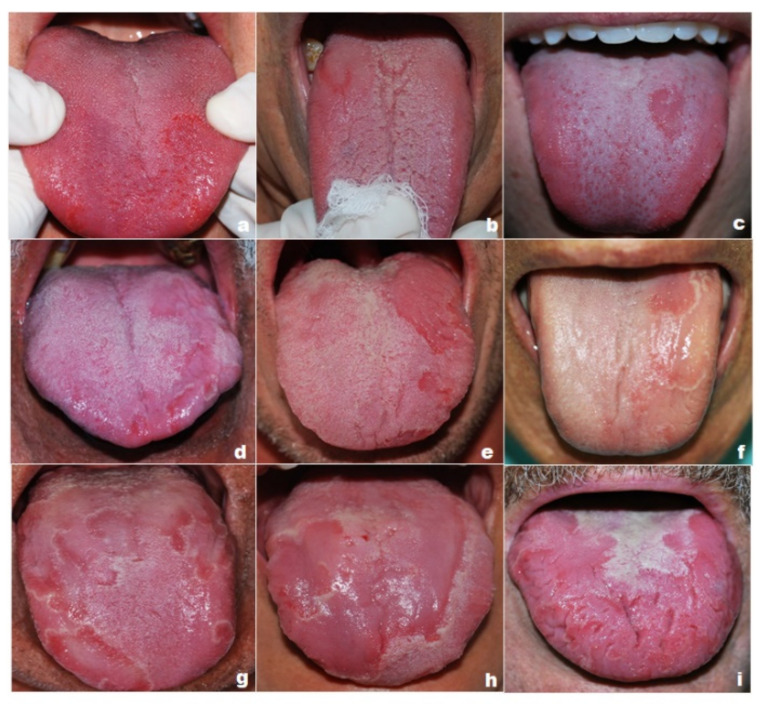
Geographic tongue categories based on clinical appearance. Nine cases of GT selected and assessed by health professionals: (**a**–**c**) Mild GT category cases. (**d**–**f**) Moderate GT category cases. (**g**–**i**) Severe GT category cases.

**Figure 3:**
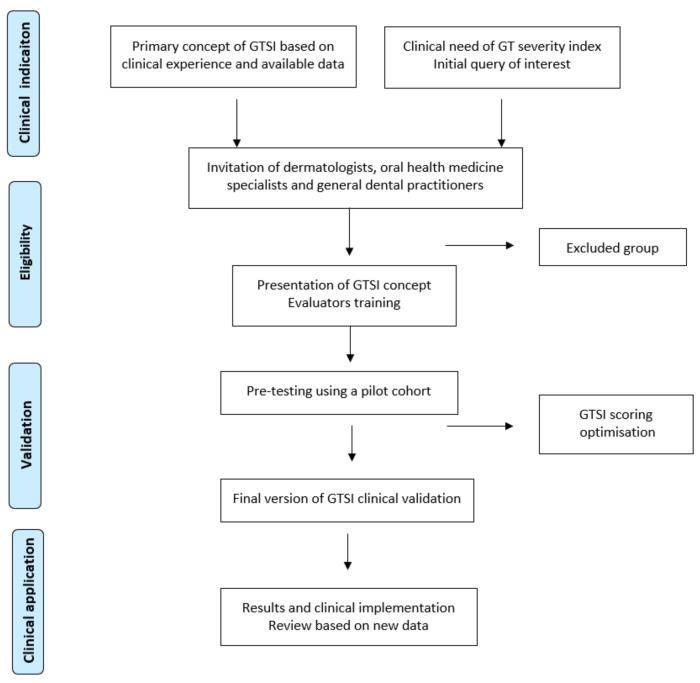
Protocol of GTASI concept development and final evaluation. Flow diagram of the GTASI establishment process based on cross-sectional expertise-driven clinical validation.

Based on previous evaluation, three concurring cases were selected from each category (mild, moderate and severe), including extreme cases and an intermediate one (Figure 2). These were sent to 51 new evaluators and clinical experts, including dentists, oral medicine specialists and dermatologists. They were given detailed information about the GT categories and protocols for selecting photographs. The structured protocol for the evaluators’ selection and GTASI validation is presented in Figure 3. The primary concept, pre-testing, GTASI validation study design, and the inclusion/exclusion criteria were initiated and managed by oral medicine team specialists. Although the chosen evaluators had different experience, they were all familiar with the clinical characteristics of GT.

In addition to photographs of the pre-selected GT cases, all evaluators received detailed instruction for assessing the severity of lesions. The GTASI was provided in an Excel spreadsheet, which was pre-filled with the index’s formulas to automatically obtain the final score and calculate each GT case’s severity for further statistical analysis and comparison.

### 2.2. Statistical Analysis

The statistical tests used in the study were performed using the Statistical Package for the Social Sciences, version 22.0. The statistical description of the studied variables was performed by proportions (when the variables were categorical) and means, standard deviations, median and minimum–maximum values (when the variables were numerical). The quantitative results of the professionals’ evaluations were compared using the non-parametric Kruskal–Wallis test to validate the clinical applicability of the new GTAS index. The statistical significance level for all analyses was *p* < 0.05.

## 3. Results

The group of evaluators that conducted the GTASI validation in clinical settings consisted of 51 professionals: 17 dentists (33%), 22 oral medicine specialists (43%) and 12 dermatologists (24%). Three GT cases from each GTASI category were sent to new evaluators and the most experienced specialists were considered as “gold standard” experts. With regard to demographic characteristics, the group comprised 48 females (94%), and age ranged from 26 to 68 years with a mean of 36 years (standard deviation = 12 years), with general dentists being the youngest and least experienced with oral lesions diagnostics. Considering overall professional experience, we observed a variation of 2 to 44 years, with a mean of 11 years (standard deviation = 11 years). The group of dermatologists was the most competent, capable, and experienced in scoring GTASI, followed by oral medicine specialists and dental surgeons.

The GTASI was found to be a feasible instrument for medical and dental professionals. Based on the score results, including both quantitative and qualitative variables, we observed significant intergroup coherence among the professionals. There were apparent intra- and inter-evaluator correlations, without a significant statistical difference between the more experienced evaluators, who assigned greater severity to GT lesions. The mild category was prevalent in this regard, while the moderate category revealed lower consistency between evaluators [13]. Mild cases of GT received a lower score compatible with the first classification of this category, with dermatologists assigning a slightly higher score compared to dentists (Table 2 and Table 3). One dermatologist assigned a high GTASI value in two cases that were considered mild, which generated a significant difference. No statistically significant difference in GTASI scoring was found in any of the quantitative data considering three different groups of evaluators/experts (Table 2, *p* > 0.05).

Moderate GT cases received scores within the expected range, although this group demonstrated significant variability in the categorisation among health professionals. In moderate GT cases 1 and 2, dentists and oral medicine specialists often scored them in the mild category. In moderate GT case 3, some dermatologists classified it as a severe clinical manifestation (Table 2 and Table 3). Severe GT cases revealed compatible scores, with less variation between professionals. Specialists, often dermatologists, assigned the highest GTASI values and dentists the lowest values (Table 2 and Table 3).

## 4. Discussion

This experts’ validation was aimed to assess the reliability of the novel clinical index GTASI in classifying GT lesions by utilising the professionals’ opinions. This was done to determine variations in the classification of GT tongue lesions. The GTASI instrument validation was accomplished by evaluating the inter-experts’ consistency and inter-rater reliability. The pre-testing of new diagnostic indices in oral medicine field carried out by clinicians with various expertise and scope of interest, is a widely approved and recommended practice for new scoring systems and their further implementation in clinical practice. The validation process consists of several stages, which are essential for index-based classifications of medical conditions that manifest with a wide range of symptoms, forms and variations. This study showed that GTASI is a valid clinical instrument that can be used by various health professionals. GTASI exhibits acceptable and sufficient reliability in the context of patients’ clinical management and research.

Although the chronic oral condition GT is considered a lesion of unknown aetiology, it is deemed to be closely associated with psoriasis [14,15,16,17,18,19]. Despite usually being asymptomatic, many patients complain of burning or itching, and they can suffer from aesthetic and/or psychological issues. Currently, there is no recommended treatment for symptomatic GT cases and no detailed clinical parameters for grading GT severity to establish an appropriate diagnostic and therapeutic protocol, if clinically justified. Here, this study utilised experts’ opinion to validate the reliability of the newly developed GTASI in assessing and classifying GT lesions based on the PASI scale [13,20]. Characteristics of clinical studies [21,22,23,24,25,26,27] containing the results of the hypothetical interrelationship between GT and psoriasis is presented in Supplementary Materials, Table S1.

The GTASI was validated by experienced clinical evaluators as a diagnostic measure to assess 100 cases of GT. Based on these results, we assessed the performance of GTASI to estimate subtle variations in the evaluation and classification of GT lesions. This was carried out by GT lesions being assessed by professionals from different specialties with unequal levels of experience. Studies showed that experience in the clinical routine of evaluating psoriatic lesions using the PASI improved the precision of the index estimation, suggesting that this factor directly relates to the evaluations performed with the GTASI [20].

To evaluate the professionals’ performance related to GT diagnosis, we carried out categorical and agreement assessments on the final scores of the different groups of evaluators. Overall, the three cases categorised as mild had a high rate of agreement between the three groups of evaluators, with overall values above 94% of correct answers in the categorical evaluation. The scores for these cases were low for the three groups. However, one dermatologist assessed two cases as being severe (scores 17 and 24 points), revealing a statistical difference between specialist dermatologists and other professionals in this category. There may have been a discrepancy in the score validation in cases that received maximum scores, while the other professionals assessed the extension with lower scores. Potentially, the professional considered the entire length of the cleft tongue in one of the cases categorised as mild (Figure 2).

Moderate cases of GT had the highest disagreement among the three groups. This was also observed amongst the experienced, “gold standard” professionals, but in smaller proportions. This disparity can also be observed in PASI evaluations, indicating that the higher the categorisation of lesions, the greater the probability of errors in their evaluation. This indicates that simplifying the categorisations and using instruments that help to level the evaluators could reduce such discrepancies in the index’s results [28,29]. Severe cases, as well as mild ones, had good agreement between the evaluators, showing that the evaluation and classification of mild or very severe lesions does not create difficulties for the professionals.

Overall, as expected, experienced dermatologists were more accurate in evaluating cases and presented the highest rates of agreement, followed by oral medicine specialists and dentists. This demonstrates that, despite not carrying out oral examinations regularly, dermatologists are able to identify GT lesions, using the GTASI as a clinical diagnostic tool to verify the occurrence of GT in psoriatic patients. Consequently, it is predicted that they provide optimal standard of care to those patients diagnosed with GT, referring to other specialists for follow-up and treatment, thus ensuring better GT management [30]. This group also assigned greater severity to GT lesions, followed by oral medicine specialists, corroborating the findings of the first assessment by experienced evaluators [13]. This fact reinforces that a specialist’s clinical experience improves the recognition of GT lesions, which provides succinct standardisation for GT classification and generates a more accurate GTASI score. On the contrary, less experienced non-specialist professionals tend to underestimate GT lesions’ severity.

It is expected that future studies, including cross-comparison evaluations and interdisciplinary negotiation will enhance and render the reproducibility of GTASI. In addition, its implementation in under- and postgraduate education, especially in the oral medicine field, would improve the recognition of characteristic features of intraoral lesions, with a suspected systemic aetiology. The proposed protocol for new index validation in clinical setting can be used for the development of comprehensive classifications of other oral manifestations, reflecting the complex nature of oral mucosa abnormalities linked to systemic conditions.

### Strengths and Limitations

Similar to the PASI, the newly proposed GTASI exhibited good reliability criteria in assessing GT, suggesting that it is a first-choice indexing instrument for determining the severity, clinical definition and follow-up of GT lesions [30,31]. Several studies support the reliability of the PASI because it provides an acceptable level of correlation and agreement in assessing psoriasis, even though it may be affected by differences in its interpretation [25,31,32,33,34,35].

Generally, dermatologists are accustomed to evaluating lesions using condition-specific indexes; however, this does not apply to dental practitioners, which impacted our findings. The wide variability and discrepancy in professional experience might be a limiting factor because the GTASI constitutes a subjective index that is directly influenced by clinical experience. In addition, based on the PASI, four levels of GT intensity were used to classify the lesions, which may have increased the subjectivity of the scoring system. It is assumed that this disadvantage could be mitigated by limiting the index to three intensity categories and introducing tools to guide the evaluation, thus promoting the evaluators’ calibration, and reducing the variation in results.

## 5. Conclusions

The newly proposed GTASI has been validated as a reliable clinical instrument, presenting a predictable coherence in the evaluation and attribution of severity to GT lesions among the different specialties. Dentists, oral medicine specialists and dermatologists showed a high level of agreement and a significant rate of correct GT diagnosis, especially among the most experienced clinical experts considering the mild and severe categories of GT. Although the GTASI seems highly reproducible and can be used in various clinical settings, further critical appraisals and experts’ agreement are required to confirm its credibility in clinical settings and in supporting clinical diagnosis protocols.

## Figures and Tables

**Table 1:** Comparison of aetiology, triggers, pathology of GT and psoriasis. Current state of knowledge.

	Geographic Tongue	Psoriasis
Aetiology	Unknown, likely inflammatory and/or autoimmune?	Autoimmune
Triggers	Food, allergy, stress nutritional deficiencies, hormonal disturbances, gluten intolerance, oral microbiota?	Infections, skin injuries, cold, stress/psychological factors?
Pathophysiology	Atrophy of filiform papillae (depapillation, loss of epithelium), intense aggregation of neutrophiles in epithelium	Epidermal hyperproliferation, parkeratosis, increased epidermal the epidermal cell turnover rate
Pathological pathways	Neutrophiles, inflammatory mediators	Pro-inflammatory cytokines, with a dominant IL-23 and Th17 axis

**Table 2:** Comparison of clinical assessment of GT severity carried out by dental surgeons, specialists in oral medicine and specialist dermatologists.

Cases	Severity	Dental Surgeon (n = 17)	Oral Medicine Specialist (n = 22)	Dermatologist (n = 12)	Total (n = 51)
Mild 1	Mild	16 (94%)	22 (100%)	11 (92%)	49 (96%)
	Moderate	1 (6%)	0 (0%)	0 (0%)	1 (2%)
	Severe	0 (0%)	0 (0%)	1 (8%)	1 (2%)
Mild 2	Mild	17 (100%)	22 (100%)	9 (75%)	48 (94%)
	Moderate	0 (0%)	0 (0%)	2 (17%)	2 (4%)
	Severe	0 (0%)	0 (0%)	1 (8%)	1 (2%)
Mild 3	Mild	16 (94%)	22 (10%)	11 (92%)	49 (96%)
	Moderate	1 (6%)	0 (0%)	1 (8%)	2 (4%)
	Severe	0 (0%)	0 (0%)	0 (0%)	0 (0%)
Moderate 1	Mild	6 (35%)	8 (36%)	3 (25%)	17 (33%)
	Moderate	8 (47%)	13 (59%)	8 (67%)	29 (57%)
	Severe	3 (18%)	1 (5%)	1 (8%)	5 (10%)
Moderate 2	Mild	13 (77%)	15 (68%)	7 (58%)	35 (69%)
	Moderate	4 (23%)	7 (32%)	6 (42%)	16 (31%)
	Severe	0 (0%)	0 (0%)	0 (0%)	0 (0%)
Moderate 3	Mild	11 (65%)	11 (50%)	7 (58%)	29 (57%)
	Moderate	5 (29%)	9 (41%)	1 (9%)	15 (29%)
	Severe	1 (6%)	2 (9%)	4 (33%)	7 (14%)
Severe 1	Mild	1 (6%)	0 (0%)	2 (17%)	3 (6%)
	Moderate	6 (35%)	7 (32%)	1 (8%)	14 (27%)
	Severe	10 (59%)	15 (68%)	9 (75%)	34 (67%)
Severe 2	Mild	1 (6%)	0 (0%)	0 (0%)	1 (2%)
	Moderate	1 (6%)	2 (9%)	1 (8%)	4 (8%)
	Severe	15 (88%)	20 (91%)	11 (92%)	46 (90%)
Severe 3	Mild	1 (6%)	2 (9%)	0 (0%)	3 (6%)
	Moderate	3 (18%)	3 (14%)	1 (8%)	7 (14%)
	Severe	13 (76%)	17 (77%)	11 (92%)	41 (80%)

**Table 3:** Quantitative evaluation of differences in GTASI scoring between experts’ groups.

Cases	Evaluators *	Mean	Median	Standard Deviation	Minimum	Maximum	*p* *
Mild 1	Dental surgeon	1.2	0.9	1.8	0	8	0.410
	Oral medicine specialist	1.2	0.8	1	0	4	
	Dermatologist	3	1.5	4.6	1	17	
	Total	1.6	0.9	2.6	0	17	
Mild 2	Dental surgeon	1.6	1.2	1.5	0	5	0.660
	Oral medicine specialist	1.8	1.3	1.5	0	6	
	Dermatologist	5.2	3	6.5	1	24	
	Total	2.6	1.3	3.6	0	24	
Mild 3	Dental surgeon	2	1.2	1.9	0	7	0.305
	Oral medicine specialist	1.3	0.8	1.2	0	4	
	Dermatologist	2.2	1.9	2.4	0	9	
	Total	1.7	1.2	1.8	0	9	
Moderate 1	Dental surgeon	8.4	6.9	5	2	21	0.480
	Oral medicine specialist	7.3	7.2	2.6	3	12	
	Dermatologist	9.3	7.8	5.2	3	24	
	Total	8.1	7.3	4.2	2	24	
Moderate 2	Dental surgeon	5	4.8	2	1	8	0.675
	Oral medicine specialist	5.8	4.8	2.5	3	11	
	Dermatologist	5.3	5	2.8	1	10	
	Total	5.4	4.8	2.4	1	11	
Moderate 3	Dental surgeon	6.1	4.5	4.7	1	21	0.439
	Oral medicine specialist	7	6.6	3.2	1	15	
	Dermatologist	7.8	3.7	6.6	1	18	
	Total	6.9	6	4.6	1	21	
Severe 1	Dental surgeon	15.94	13.9	8.55	3	36	0.929
	Oral medicine specialist	15.45	15.25	5.61	7	26	
	Dermatologist	15.83	17.4	6.39	6	27	
	Total	15.7	15.5	6.75	3	36	
Severe 2	Dental surgeon	22.41	23.6	8.75	5	37	0.941
	Oral medicine specialist	21.9	21.3	7.76	8	35	
	Dermatologist	22.33	22.15	8.02	7	36	
	Total	22.15	22.2	8	5	37	
Severe 3	Dental surgeon	13.94	13	5.24	3	27	0.597
	Oral medicine specialist	16.22	15	8.25	4	37	
	Dermatologist	15.33	13.9	3.98	11	25	
	Total	15.18	14	6.47	3	37	

(*)—statistical significance with *p* < 0.05.

## Data Availability

Collected data available on request.

## Supplementary Materials

The following are available online at https://www.mdpi.com/article/10.3390/jcm10235493/s1, Table S1: Main characteristics of studies investigating the relationship between psoriasis and geographic tongue.
