# Supplementary material for: Applicability of the Geographic Tongue Area and Severity Index among Healthcare Professionals: A Cross-Sectional Clinical Validation of a Newly Developed Geographic Tongue Scoring System
Source: J Clin Med. 2021 Nov 24;10(23):5493. doi: 10.3390/jcm10235493 (PMC8658609; doi:10.3390/jcm10235493)
Supplement: Supplementary file 1 [file jcm-10-05493-s001.zip › jcm-1406199-supplementary.pdf]

Table S1. Main characteristics of studies investigating the relationship between psoriasis and geographic tongue.

Authors, year	Study design	Results	Conclusions
Pogrel *et a.l*, 1988 [16]	Cross-sectional	100 patients with acute relapse of cutaneous psoriasis present intraoral lesions, include benign migratory glossitis and ectopic geographic tongue in 24 cases.	Despite the rarity of oral psoriatic lesions, there does appear to be a significantly increased incidence of geographic tongue and ectopic geographic tongue. It's suggested that may represent true oral manifestations of psoriasis.
Gonzaga *et al.*, 1996 [12]	Case-control	The association with HLA-Cw6 was present in 59.1% of the patients with psoriasis, in 43.8% of the patients with geographic tongue, and in only 12.6% of the controls.	The primary association of HLA-Cw6 with geographic tongue is strongly in favour of the concept that, at least, some cases of geographic tongue may represent a true manifestation of psoriasis.
Daneshpazhooh *et al.*, 2004 [20]	Case-control	Benign migratory glossitis was significantly more frequent in psoriatic patients (28 cases, 14%) than the control group (12, 6%). In 12 patients (6%) fissured tongue and benign migratory glossitis coexisted. In the control group, fissured tongue and benign migratory glossitis coexisted in 2 cases (1%).	Although oral lesions might not be considered authentic oral psoriasis unless proven histologically and with a parallel clinical course, nonspecific tongue lesions are significantly more frequent in psoriatic cases.
Zargari *et al.*, 2006 [21]	Prospective	306 patients with psoriasis were enrolled in the study. Geographic tongue was seen in 17 patients (5.6%) and 5 patients (1.6%) had both fissured tongue and geographic tongue. The rate of geographic tongue was found to be higher in early onset than in late-onset psoriasis (7.2% vs. 1.3%).	From analysis of this study, it appears that GT but not FT is more common in early onset psoriasis and may be an indicator or predictor of severity of the disease
Costa *et al.*, 2009 [22]	Case-control	The most prevalent oral lesions recorded in 166 patients with psoriasis was fissured tongue (34,3%) and geographic tongue (18,1%). In the control group (n=166), fissured tongue was observed in 16.2% and geographic tongue in 4.2% of patients.	Psoriatic patients presented no specific oral lesion different from those seen in the control group. In a strictly statistical finding, fissured tongue and geographic were seen to occur with greater prevalence in patients with psoriasis than in the control group.
Singh *et al.*, 2013 [11]	Case-control	Significantly, more number of patients had oral lesions compared to controls. There was a strongly significant association between psoriasis and migratory glossitis. PASI was significantly more in patients who had migratory glossitis compared to those who did not.	The results suggest that migratory glossitis is a rare manifestation of psoriasis which occurs more commonly in male patients and in those with severe disease.
Picciani *et al.*, 2019 [19]	Cross-sectional	The immunohistochemical analysis was directed to theimmunoreactivity of antibodies anti-IL-6, anti-IL-17, andanti-IL-23 in both GT and psoriasis. This analysis evidenced similarity in the	This study shows that the inflammatory response and immunostaining of IL-6, IL-17, and IL-23 are often similar in both geographic tongue and psoriasis, reinforcing the possibility that there is a type of geographic tongue that represents an oral manifestation of psoriasis.

		distribution and quantity of immunopositive cells in both skin and oral lesions.	
Costa et al., 2020 [23]	Case-control	Individuals with psoriasis (n=295) showed significantly more oral lesions (24,7%) than controls (8,6%). Higher occurrence of geographic tongue was observed in individuals with psoriasis (5%) than control group (0,55%).	This study demonstrates that psoriatic individuals had a higher frequency of geographic tongue than controls. Additionally, worse subjective oral health indicators scores in frequency and severity are observed in psoriatic individuals with oral lesions.
Olejink *et* al., 2020 [24]	Observational case-control	The most commonly found pathologic changes in patients with psoriasis included: fissured tongue (49.6%), white coated tongue (31.5%) and geographic tongue (17.3%). However, geographic tongue was least prevalent in the group managed with the biological drugs.	This study showed a higher incidence of tongue lesions in patients with psoriasis, including fissured, geographic, and coated tongue. The type of te treatment seems to affect the prevalence of oral lesions. Geographic tongue significantly lower prevalence in the group managed with biologicals may require further investigation
